# Congenital Hydro-Cerebral Hernia

**Published:** 1858-01

**Authors:** H. P. Ayres

**Affiliations:** Fort Wayne, Indiana


ART. V.—*Congenital Hydro-Cerebral Hernia; Death; Post-mortem Examination.* By H. P. AYRES, M.D., of Fort Wayne, Indiana.

I USE the term Hydro-Cerebral Hernia for want of a better to designate the following case. Such cases may be of common occurrence, but not in my experience. The disease is different from hernia cerebri, or encephalocele, being more nearly allied to hydrocephalus externus, yet materially differing in its anatomical characters. Cases somewhat similar are on record, but their occurrence *in utero* and the results of *post-mortem* examination have been neglected.

Mrs. H., aged thirty-two, fifth pregnancy; of a good constitution. In her fourth month she found that she was more than ordinarily enlarged, and had a strangely severe pain in her left side. The extraordinary enlargement continued to increase, which ultimately rendered her almost helpless. The pain in the side became paroxysmal, was greatly increased by change of position, and sometimes insupportable.

Purgative medicines were administered with the hope of relief, but the result was only temporary. Venesection was resorted to, but with little better results than attended the administration of purgatives.

Her previous labors had been very easy, and not tedious.

Her labor in this confinement commenced at the full period of gestation. I found the labor progressing rapidly. The membranes broke soon after my arrival, and the usual amount of liquor amnii escaped. In touching, I found the head presenting, and in the course of a few minutes I felt what appeared to be another membrane. During each expulsive pain, the latter was pressed against the superior strait, but could not engage on account of its size; consequently, when the pain subsided, it would rebound. I was satisfied the membranes proper had been ruptured—that the liquor amnii had escaped—that the head had presented, but what this was, was to me a mystery. Was it a second fœtal membrane? That was impossible, for the head had surely presented. Was it a polypus? The impression made upon the finger contra-indicated such a conclusion. I felt satisfied the labor could not be accomplished, as the periphery of the presenting body forbid such an idea. I satisfied myself that fluid was contained in the sac, and hence determined to puncture it, which I did. The gush of fluid was terrible. After permitting eight or ten pints to escape, I closed the vagina, to ascertain the precise character of the fluid. Finding it to be a serous, limpid one, and that the pulse of my patient was good, I permitted the remainder to escape.

The labor was soon completed, and the difficulty became apparent.

There was an apron, or duplicature of the scalp, falling from the occipital portion of the head, and parallel with the occipital protuberance. The length of this attachment or duplicature on the head was three and a half inches, widening, as it descended, in a wedge or balloon-like sac, until it rested on the back.

On the presenting portion, or the point where the puncture was made, there was a circular space of three inches, which had the appearance of having been in close contact with the parietes of the womb, and there retained by suction, which may account for the excruciating pain felt by the mother during gestation. In every other respect the child was well formed and healthy. The next day there appeared to be a line of demarcation near the connection of the sac with the head, embracing the whole circumference of the sac, and had the appearance of sloughing. The succeeding day this line began to disappear, and a free and healthy circulation was established. The puncture closed, and the fluid began to accumulate, which evidently gave great pain to the infant. The puncture was reopened, and several ounces of a serous, limpid fluid escaped. Oleaginous and astringent dressings were applied, which greatly reduced the size of the sac. During the period of twenty-four hours, five or six ounces of fluid would accumulate, giving evident pain, which was immediately relieved when the fluid was evacuated. The child appeared entirely well, nursed heartily, looked about as infants do, and manifested all the healthy functions of the brain.

The sixteenth day it was feverish; from the seventeenth to the twentieth, it was comatose, and became greatly emaciated; on the twentieth day it died, evidently from cerebral trouble.

*Post-mortem.*—The sac was found to be of a uniform thickness with, and presented all the characteristics of the integuments of the head, and was formed by a duplication of the scalp. The pia mater and dura mater were continuous from the internal table of the cranium, diverged from the foramen in the occipital bone, and lined the sac throughout. The perforation or foramen in the bone was oval, one and a fourth inches transversely, one and three-fourths inches perpendicularly; the edges were rounded, smooth, and evidently a finished formation. A cone-shaped portion of the brain projected through the foramen one and a half inches, and was entirely healthy. The fluid, as I have remarked, was of a serous, limpid character, and was secreted in quantities varying from two to five ounces in twenty-four hours.

The character and locality of the foramen renders it highly probable that the hernial character of the trouble must have occurred before, or simultaneously with ossification.

